# A Metabologenomic approach reveals alterations in the gut microbiota of a mouse model of Alzheimer’s disease

**DOI:** 10.1371/journal.pone.0273036

**Published:** 2022-08-24

**Authors:** Francesco Favero, Elettra Barberis, Mara Gagliardi, Stefano Espinoza, Liliana Contu, Stefano Gustincich, Francesca Boccafoschi, Chiara Borsotti, Dmitry Lim, Vito Rubino, Flavio Mignone, Edoardo Pasolli, Marcello Manfredi, Silvia Zucchelli, Davide Corà, Marco Corazzari

**Affiliations:** 1 Department of Translational Medicine (DIMET) & Center for Translational Research on Autoimmune and Allergic Disease (CAAD), University of Piemonte Orientale, Novara, Italy; 2 Department of Health Science (DSS) & Center for Translational Research on Autoimmune and Allergic Disease (CAAD), University of Piemonte Orientale, Novara, Italy; 3 Department of Health Science (DSS), University of Piemonte Orientale, Novara, Italy; 4 Central RNA Laboratory, Istituto Italiano di Tecnologia, Erzelli, Genova, Italy; 5 Department of Pharmaceutical Sciences (DSF), University of Piemonte Orientale, Novara, Italy; 6 Department of ‘Studi per l’Economia e l’Impresa’ (DISEI), University of Piemonte Orientale, Novara, Italy; 7 Department of Science and Technological Innovation, University of Piemonte Orientale, Alessandria, Italy; 8 SmartSeq s.r.l., Spin-Off of the University of Piemonte Orientale, Alessandria, Italy; 9 Department of Agriculture, University of Naples Federico II, Naples, Italy; 10 Department of Health Science (DSS), Center for Translational Research on Autoimmune and Allergic Disease (CAAD) & Interdisciplinary Research Center of Autoimmune Diseases (IRCAD), University of Piemonte Orientale, Novara, Italy; University of Kansas, UNITED STATES

## Abstract

The key role played by host-microbiota interactions on human health, disease onset and progression, and on host response to treatments has increasingly emerged in the latest decades. Indeed, dysbiosis has been associated to several human diseases such as obesity, diabetes, cancer and also neurodegenerative disease, such as Parkinson, Huntington and Alzheimer’s disease (AD), although whether causative, consequence or merely an epiphenomenon is still under investigation. In the present study, we performed a metabologenomic analysis of stool samples from a mouse model of AD, the 3xTgAD. We found a significant change in the microbiota of AD mice compared to WT, with a longitudinal divergence of the F/B ratio, a parameter suggesting a gut dysbiosis. Moreover, AD mice showed a significant decrease of some amino acids, while data integration revealed a dysregulated production of desaminotyrosine (DAT) and dihydro-3-coumaric acid. Collectively, our data show a dysregulated gut microbiota associated to the onset and progression of AD, also indicating that a dysbiosis can occur prior to significant clinical signs, evidenced by early SCFA alterations, compatible with gut inflammation.

## Introduction

In the last decades, a revolution has emerged in the study, understanding, and interpretation of the role of microorganisms that intimately interact with our body, the microbiota. In particular, those who reside permanently in the gastrointestinal tract (intestinal microbiota) are now considered to participate in the biochemical and physiological activities of the human body as an ‘additional body organ’ [1]. Indeed, gut microbiota consists of trillions of microorganisms ranging from bacteria to archaea, fungi, protozoa, and viruses [1] with the total number of those organisms being extremely higher than the total number of cells in the human body. Intestinal microbiota provides protective, structural, and metabolic functions, although a full comprehension of the activities of this highly complex ecosystem is still under investigation [2]. Consequently, it appears as an essential organ influencing human health and pathological processes, such as inflammation, infections, and tumors [2].

It is important to note that although confined to a specific location in the body, the gastrointestinal (GI) tract, this additional organ is able to interact effectively not only with specific tissues of the GI, such as the intestinal epithelium, but it actively affects other distant organs, such as the central nervous system, in the so-called ‘gut-brain axis’, bidirectionally. Indeed, the involvement of the gut microbiota in neurological disorders such as, but not limited to, autism is now widely accepted [3].

Among healthy subjects there is a high variability in the composition of the intestinal microbiota; established early in life, within the first 2–3 years of the child’s life, it remains relatively unchanged for the rest of the human lifespan [4–7]. Similarly to other organs, a malfunction or dysregulation of the intestinal microbiota has a strong impact on the functions of the whole organism. However, although dysbiosis has been evidenced in several human diseases, such as obesity, coronary heart disease, diabetes, and inflammatory bowel disease [8], the key question is still: is gut dysbiosis a cause (or a contributing cause) or consequence of these disorders? There is no simple answer to this question, and it may depend on the specific disease. Therefore, it is very important to characterize the extent of dysbiosis, as this could be useful for adopting alternative strategies for disease management.

The role of the gut microbiota in neurodegenerative disease is an emerging research field. Recently, Parkinson’s disease patients showed a different gut microbiota than healthy controls [9], and growing evidence also points to gut microbiota dysbiosis in Alzheimer’s disease (AD) patients and mouse models [10–12]. A deep characterization of the gut microbiota and metabolites, such as short fatty acids and amino acids, will help in a better understanding of the etiopathology of AD. Thus, using a recently introduced metabologenomic approach [13] we analyzed complex intestinal microbial ecosystems in a deterministic (3xTgAD) mouse model of familial AD.

## Materials and methods

### Animals

Homozygous 3xTgAD mice (B6;129-Psen1tm1Mpm Tg(APPSwe,tauP301L)1Lfa/Mmjax1; n = 4) were compared to WT controls (B6129SF2/J; n = 4). WT and AD mice were co-housed at weaning (until the end of the experiments), to minimize intrinsic microbiota composition diversity, and food and water were provided ad libitum. Fecal samples were freshly collected at weaning (T0), at 2 (T1) and 6 (T2) months, placed on dry ice until sampling was complete, and immediately stored at -80°C.

The triple-transgenic (3xTgAD) mouse strain is a widely used and represents a valuable model to study AD-related molecular mechanisms because it develops both Aβ and tau brain pathology [14]. This strain exhibits age-dependent neuroanatomical and cognitive parallels to AD, including Aβ plaque formation, neurofibrillary tangles, and memory deficits, making it relevant to AD in humans. Intracellular Aβ accumulation is detected in brain regions as early as 3–4 months of age. Extracellular Aβ deposits appear by six months in the frontal cortex and become more extensive by twelve months. Synaptic transmission and long-term potentiation (LTP) are impaired by 6 months, and hyper-phosphorylated tau is detected at 12–15 months [14]. All mice were housed in autoclaved cages (2 WT+2AD/cage) in a specific pathogen free (SPF) Animal Facility (at IIT, Genova, IT), with twelve-hour light/dark cycles (i.e., light from 7:00 am to 7:00 pm). All procedures were approved by the local Ethics Committee for Animal Welfare (IACUC No 1766AA.33 - 748/2017-PR) and conformed to the European Community regulations for animal use in research (2010/63 UE).

### Metagenomics

The metagenomic analysis was performed by the ‘Metagenomic’ facility, and data analysis was performed by the ‘Bioinformatics’ facility at CAAD (University of Piemonte Orientale, Novara, IT).

#### DNA isolation

Fecal samples were thawed at room temperature and microbial DNA was isolated using the QIAmp® PowerFecal® Pro DNA isolation kit (Qiagen), according to the manufacturer’s instructions. The yield and quality of bacterial DNA was determined by a NanoDropTM 2000 spectrophotometer (Thermo Fisher Scientifics Inc). The quantity was assessed using an InvitrogenTM QubitTM 1X dsDNA HS Assay Kit (Invitrogen) and a Qubit 4 fluorometer (Invitrogen).

#### 16S gene sequencing

Purified DNA samples were subjected to 16S rRNA metagenomics analysis to compare the distribution and relative or absolute abundance of microbial consortia in the original fecal samples at the relevant time points. Analysis was performed using the Microbiota Solution B Kit, a Next Generation Sequencing (NGS) in vitro molecular test, CE-IVD marked. Polymerase chain reaction (PCR) amplification of the V3-V4-V6 hypervariable regions of bacterial 16S rRNA was obtained by employing degenerated primers, according to the manufacturer’s instructions. PCR products were purified using Agencourt AMPure XP magnetic beads (Beckman Coulter Inc., Brea, CA, USA) and indexes were added in a subsequent step. The hypervariable V3-V4-V6 regions of the bacterial 16S rRNA were amplified according to the manufacturer’s instructions.

The DNA concentration of the libraries was measured fluorometrically and samples were pooled in equimolar concentrations. The final 16S rRNA amplicon libraries were sequenced on a MiSeq Illumina® sequencing platform (Illumina, CA, USA) using a MiSeq Reagent Kit v2 Nano cartridge for a 2x250 paired-end sequencing.

The amplicon length (840 bp) provided an average coverage of 60,482 total reads per sample (range 25,460–120,886).

*Analysis of 16S rRNA gene sequences*. Raw 16S rRNA reads have been analyzed using Microbat, a tool for 16S reads taxonomic assignment developed by SmartSeq, a spin-off of the Piemonte Orientale University. The resulting OTU table of Microbat reporting relative abundance values of each taxa was used to produce pie charts at phylum taxonomic level. Resulting OTU table of Microbat containing raw reads counts assigned to each taxa was then filtered on a reads number cutoff ≥ 5 in at least one sample, while unclassified OTUs have been discarded in all the subsequent analysis. The resulting filtered OTU table was then processed using the webtool Microbiomeanalyst [18] with the following parameters (default where not specified):

Data Filtering: No low count filter; No variance filter.

Data Normalization: no Data rarefying; Data scaling = Total Sum scaling; Data transformation = Do not transform my data.

Abundance Profiling: data are shown at “Family” taxonomic level. Taxa resolution: All taxa are shown, small taxa have not been merged.

Alpha Diversity Profiling & Significance Testing: Diversity measure = Shannon; Statistical Method = Mann-Withney/Kruskal-Wallis.

Beta Diversity Profiling & Significance Testing: Distance method = Jensen-Shannon Divergence; Statistical method = PERMANOVA.

Differential Abundance Analysis Methods (RNA-Seq methods): Algorithm: DESeq2; Adjusted P.value cutoff = 0.1 and |log2FC| > 1.

Hierarchical Clustering & Heatmap visualization: Distance measure = Pearson; Clustering Algorithm = Complete.

Relative Abundance graph was calculated using MicrobiomeAnalyst tool “stacked bar/area plot”.

For longitudinal investigations, only differential taxa present in AD samples have been considered interesting, while differential taxa present also in the same longitudinal comparison with the same trend in WT samples have been filtered out. Differential taxa have been subsequently plotted with Heatmap Clustering function of MicrobiomeAnalyst.

#### Metabolomics

Metabolomic analysis was performed by the ‘Metabolomics & Proteomics’ facility at CAAD (University of Piemonte Orientale, Novara, IT).

Methyl tert-butyl ether (MTBE), N,O-Bis(trimethylsilyl)trifluoroacetamide (BSTFA), methoxamine, hexadecane, propanoic acid d2 and tridecanoic acid were purchased from Merck (Darmstad, Germany). Pyridine, water and acetonitrile were from VWR (Milano, Italy). Methanol and isopropanol were from Scharlab (Barcelona, Spain). Commercial mix standard of free fatty acids including acetic acid, propanoic acid, butanoic acid, isobutyric acid (propanoic acid, 2-methyl), isovaleric acid (butanoic acid, 3-methyl) and valeric acid (pentanoic acid) were from Restek (Bellefonte, PA).

SCFAs were extracted from 30 mg of fecal sample. Briefly, 300 μL of water and 16 μL of the internal standard propanoic acid d2 (20,5 ppm) and 5 μL of tridecanoic acid (500 ppm) were added. The sample was then homogenized with FastPrep-24 5G (MP Biomedicals, USA) for 40 s at 6.0 m/s and vortexed at 1000 rpm at 4°C for 30 minutes. The sample centrifuged at 21.1 x g for 30 minutes at 4°C. 100 μL of supernatant were placed in a new tube, bringing the pH to 2 using 6 M HCl. For the extraction of SCFAs 140 μL of MTBE were added and the tube was placed on a rotator for 15 minutes at 40 rpm, followed by a centrifugation for 10 minutes at 4°C at 21.1 x g. Then, 100 μL of the organic phase, that contains the SCFAs, were analyzed with bi-dimensional gas chromatography coupled to mass spectrometry (GCxGC-TOFMS). The remaining water phase solution was then subjected to a second extraction described in the following paragraph. Calibration curve for the absolute quantification of the main SCFAs were also built [15,16].

The remaining aqueous phase, that contains other small molecules such as amino-acids, sugars, medium and long fatty acids, was extracted again using methanol-isopropanol-acetonitrile. Briefly, 100 μL of water from the first liquid-liquid extraction were subjected to a second extraction using 1 mL mixture of acetonitrile (ACN), methanol (MeOH) and water 3:3:2. The sample was vortexed for 15 seconds and centrifuged for 15 minutes at 20°C at 14.5 x g. One mL of the supernatant was placed in a new tube and dried in a speed-vacuum and then placed at -20°C until derivatization. The sample was finally derivatized with methoximation (20 μL of methoxamine, 80°C, 20 min) and silylation (90 μL of N,O-Bis(trimethylsilyl)trifluoroacetamide, 80°C, 20 min) prior the GCxGC-TOFMS analysis [17].

*GCxGC-TOFMS analysis*. For the analysis, a LECO Pegasus BT 4D GCxGC-TOFMS instrument (Leco Corp., St. Josef, MI, USA) equipped with a LECO dual stage quad jet thermal modulator was used. The GC part of the instrument was an Agilent 7890 gas chromatograph (Agilent Technologies, Palo Alto, CA), equipped with a split/splitless injector. The first dimension column was a 30 m Rxi-5Sil (Restek Corp., Bellefonte, PA) MS capillary column with an internal diameter of 0.25 mm and a stationary phase film thickness of 0.25 μm for metabolite from aqueous solution, while for SCFAs analysis the column was a 30 m DB-FATWAX-UI (Agilent Technologies, Santa Clara, CA) with a diameter of 0.25 mm and a film thickness of 0.25 μm, and the second dimension chromatographic columns was a 2 m Rxi-17Sil MS (Restek Corp., Bellefonte, PA) with a diameter of 0.25 mm and a film thickness of 0.25 μm. High-purity helium (99,9999%) was used as the carrier gas with a flow rate of 1.4 mL/min. 1 μL of sample was injected in splitless mode at 250°C. The temperature program for metabolites analysis was as follows: the initial temperature was set at 70°C for 2 minutes, then ramped 6°C/min up to 160°C, 10°C/min up to 240°C, 20°C/min to 300 and then held at this value for 6 minutes. The secondary column was maintained at +5°C relative to the GC oven temperature of the first column. The temperature program for SCFAs was as follows: the initial temperature was 40°C for 2 minutes, then ramped 7°C/min up to 165°C, 25°C/min up to 240°C, maintained for 5 minutes. The secondary column was maintained at +5°C relative to the GC oven temperature of the first column. Electron impact ionization was applied (70 eV). The ion source temperature was set at 250°C, the mass range was 40 to 300 m/z with an extraction frequency of 32 kHz, for the SCFAs analysis, while for the metabolites the mass range was 25 to 550 m/z. The acquisition rates were 200 spectra/s. The modulation periods for both programs were 4s for the entire run. The modulator temperature offset was set at +15°C relative to the secondary oven temperature, while the transfer line was set at 280°C Chromatograms were acquired in TIC (total ion current) mode. Peaks with signal-to-noise (S/N) value lower than 500.0 were rejected. ChromaTOF version 5.31 was used for raw data processing. Mass spectral assignment was performed by matching with NIST MS Search 2.3 libraries adding Fiehn Library. Commercial mix standard of free fatty acids composed by acetic acid, propanoic acid, propanoic acid 2-methyl, butanoic acid, butanoic acid 3-methyl and pentanoic acid was run individually and EI spectra were matched against the NIST library. The calibration curves of the SCFAs were obtained using excels, while the analytical results obtained from untargeted metabolomics were processed and compared with the opensource software MetaboAnalyst 5.0 (www.metaboanalyst.org). Data were integrated using M2IA opensource web server (http://m2ia.met-bioinformatics.cn/).

*Statistical analysis*. Alpha diversity statistical analysis was performed using Mann-Withney/Kruskal-Wallis with MicrobiomeAnalyst web tool. Beta diversity statistical significance was measured using PERMANOVA statistical test again in MicrobiomeAnalyst web tool. Statistically different taxa have been individually tested using DESeq2 algorithm using |log2FC|>1 and padj <0.1 as cut-offs.

Firmicutes/Bacteroidetes ratio statistically significance has been calculated using ANOVA with GraphPad software.

Statistical analysis of metabolomic data was performed through MetaboAnalyst 5.0. No data filtering was applied to raw data. The areas from untargeted analysis were normalized by the sum and then mean-centered, while no more data transformation have been performed. The hierarchical cluster analyses heat map was calculated using Euclidean distance and Ward’s linkage. Changes between two groups of samples were calculated with unpaired fold change (FC) analysis, computing the ratios between two groups means. FC threshold of 1.3 has been set to discriminate significative changes between the two groups, while the statistical significance was calculated using parametric t-test with GraphPad software. The molecules with a p-value threshold less than or equal to 0.05 have been selected as possible markers.

Metabolo-genomics data integration was performed using M2IA. The functional network analysis was based on Weighted gene co-expression network analysis (WGCNA) algorithm, chosen to collapse co-abundant metabolites into different clusters. The final correlation between metabolite clusters and microbial functional modes with phenotype information was investigated using Spearman correlation.

## Results

**Fig 1:**
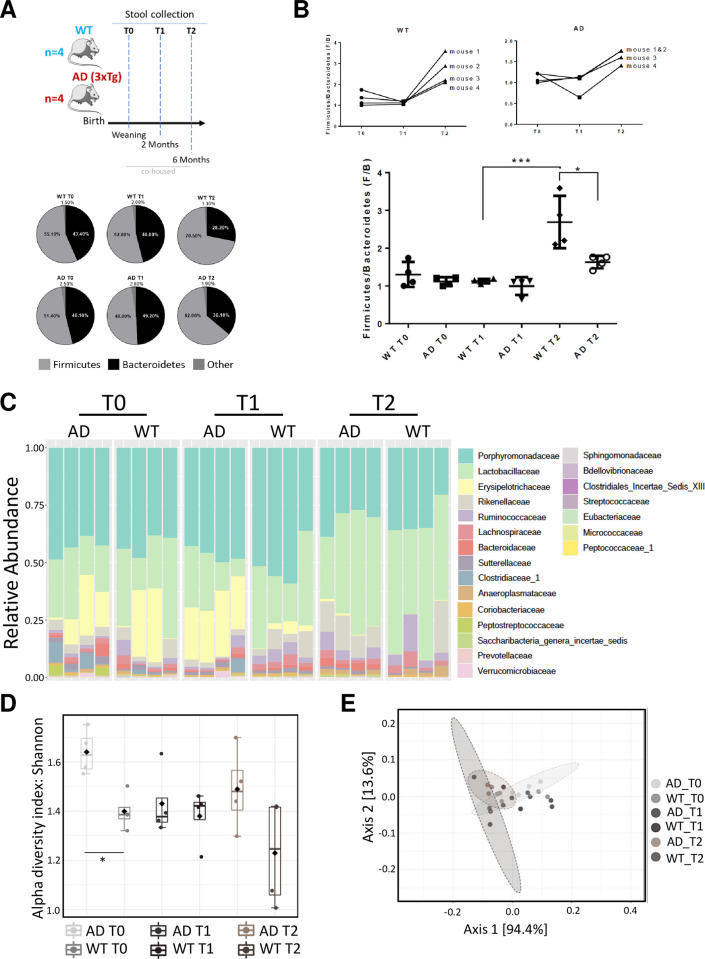
Global 16S metagenomics profiling. A) Schematic representation of study design (upper panel); Abundance profiles of microbes (percentage) in mice stool at the phylum taxonomic rank (bottom panel); samples were named as reported in experimental design. B) The Firmicutes/Bacteroidetes ratio was calculated in each animal at the indicated time point T0, T1 and T2, and plotted individually (upper panels) or grouped (bottom panel). An ANOVA analysis was performed using all groups. *p<0,05; *** p<0,001. It is important to note that a statistical difference was only observed compared WT T1 vs WT T2 groups, and WT T2 vs AD T2 groups. C) Relative abundance profiles of microbes in mice stool at family taxonomic rank. D) Boxplots showing alpha diversity (Shannon index) values across experimental conditions at family taxonomic rank. E) PCA plot based on Jensen-Shannon divergence values across experimental conditions at family taxonomic rank. * p<0.05.

Here, we sought to study the overall composition of the microbiota in a mouse model recapitulating the progression of Alzheimer’s disease, the 3xTgAD mice, compared with matched WT controls. Metagenomics and metabolomics were used to deeply characterize the alterations of microbial species and small molecules in the gut. Fig 1A (upper panel) shows our experimental layout, in which animals were maintained in a specific pathogen free animal facility and stools from 4 wild type (WT) and 4 3xTgAD mice were collected at weaning (T0), 2 months (T1) and 6 months (T2).

### Metagenomic analysis of the gut microbiome composition in the 3xTgAD mouse model of Alzheimer’s disease

The microbiota composition was characterized by means of 16S sequencing, with analysis performed at different taxonomical levels, allowing for a complete dissection of the full microbiota according to a variety of parameters. DNA from each sample was extracted and sequenced as reported in the Materials and Methods section.

We then evaluated the relative abundance of each taxa in each sample, at the phylum level (pie charts shown in Fig 1A, bottom panel). As expected, results highlighted a major contribution of both Firmicutes (F) and Bacteroidetes (B) in the composition of the microbiota, at all time points evaluated, in both WT and AD mice with a similar F/B ratio trend at T0 (1.2 WT vs 1.1 AD) and T1 (1.1 WT vs 1.0 AD), comparing WT and AD. Interestingly, while a physiological increase in the F/B ratio was evidenced in both groups of animals at T2, a reduced increase in the AD group at 6 months (2.6 WT vs 1.7 AD) indicates a lower per-centage of Firmicutes, compared to WT littermates (ANOVA one-way, p<0.05, Fig 1B). Moreover, an increase in the Candidatus Saccharibacteria phylum was also found in the microbiota of AD animals at T2 (0.002%±0.127% WT vs 0.258%±0.100% AD; DESeq2 statistical test: log2FC = 7.926, FDR = 2.20E-8), together with an increased presence of Proteobacteria (0.220%±0.059% WT vs 0.944%±0.183% AD), although not statistically significant.

In addition, microbiota alterations were in depth investigated by analyzing the data at the family taxonomic rank. Fig 1C shows a stacked bar chart representing the relative abundance of each family across samples, at each time point. This analysis revealed a very heterogenous presence of Erysipelotrichaceae (Firmicutes phylum) in both WT and AD mice at weaning (15.59%±17.45% and 13.42%±10.52% respectively), with a consistent decrease that was observed at both 2 (DESeq2 statistical test: log2FC = -3.3074, FDR = 0.096 WT-T0 vs WT-T1) and 6 (DESeq2 statistical test: log2FC = -2.787, FDR = 0.008 WT-T1 vs WT-T2) months in WT mice (2.73%±2.13% WT-T1 and 0.25%±0.12% WT-T2, respectively). On the contrary, an increased trend in the presence of this family was evident at 2 months in AD mice (although not statistically significant) with a decrease observed at 6 months (13.42%±10.52 AD-T0, 24.29%±2.97% AD-T1, 0.70%±0.46% AD-T2; DESeq2 statistical test: log2FC = -6.469, FDR = 1.51E-15 in AD-T1 vs AD-T2), but still higher compared to WT (0.70%±0.46% AD-T2 vs 0.25%±0.12% WT-T2; although not statistically significant). In parallel, the Bacteroidetes family of Porphyromonadaceae appears to be predominant (about 50%) at both T0 and T1 in AD and WT samples, while decreasing at T2, with a similar trend in both AD and WT.

On the contrary, Lactobacillaceae (Firmicutes) show an increased trend at T2 in both AD and WT (at a similar extent), compared to both T0 and T1 (Fig 1C).

To better understand the differences among samples, we then evaluated two statistical parameters commonly used to address the variability in metagenomic samples: the alpha and beta diversity. Alpha diversity at family taxonomic level across samples was calculated with the Shannon index and reported in Fig 1D. This analysis evidenced a significant different biodiversity of WT and AD samples at T0 (Mann Whitney test: p-value <0.05), while becoming similar at T1, and indicating a divergent trend at T2.

Fig 1E represents a PCA plot showing the beta diversity across samples at family taxonomic level indicating differences among the experimental groups (Permanova test: p-value < 0.05); colored ellipses highlight groups that will undergo further analyses at genus level.

**Fig 2:**
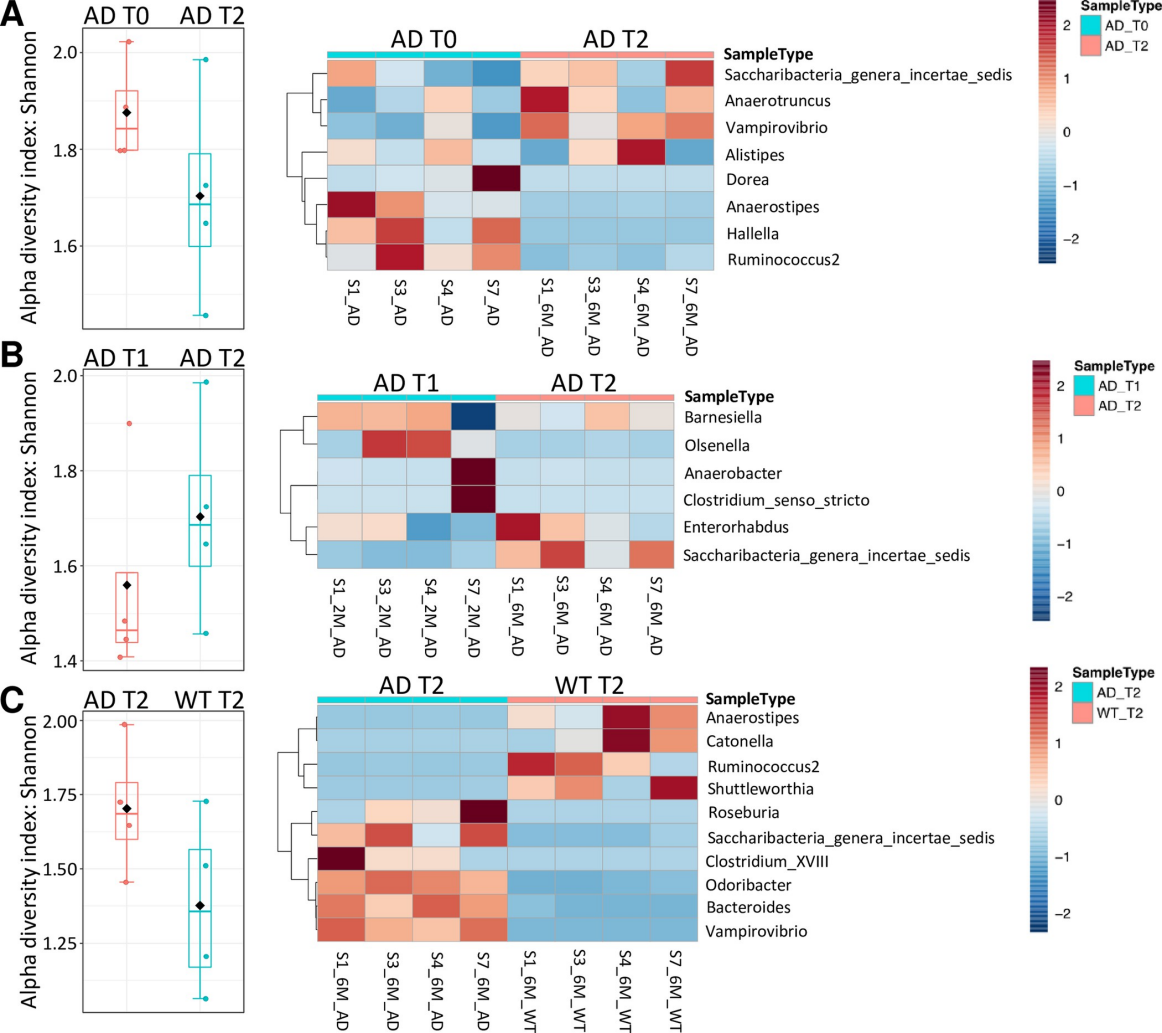
AD progression drives global perturbations in the microbiota. A) Differences at the genus taxonomic rank in mice stool microbiota between T2 and T0 time points, for AD samples. Plots show alpha diversity (Shannon index) for the samples considered and the corresponding heatmap of the differentially modulated genera. B) Differences at the genus taxonomic rank in mice stool microbiota between T1 and T2 time points, for AD samples. Plots show alpha diversity (Shannon index) for the samples considered and the corresponding heatmap of the differentially modulated genera. C) Differences at the genus taxonomic rank in mice stool microbiota between WT samples and AD samples at T2 time points. Plots show alpha diversity (Shannon index) for the samples considered and the corresponding heatmap of the differentially modulated genera.

Stemming from these considerations, we moved our attention to the Genus level, and we particularly investigated possible modulation in the microbiota of AD mice, among the following three comparisons: T0 versus T2 (Fig 2A), T1 versus T2 (Fig 2B), and AD vs WT at T2 (Fig 2C).

Data reported in Fig 2 summarizes the main results of these comparisons. The left boxplots panels show the alpha diversity at the genus level for the indicated samples, calculated with the Shannon index, while the right panels show unsupervised hierarchical clustering of only differential genera among the respective comparisons (AD-T0 vs AD-T2 –Fig 2A–or AD-T1 vs AD-T2 –Fig 2B, or AD-T2 vs WT-T2 –Fig 2C). In the heatmaps (right panels), data were corrected by subtracting the genera showing the same trend in both WT and AD samples. Different genera have been calculated using DESeq2 RNA-Seq approach with MicrobiomeAnalyst (padj <0.1 |log2FC| >1) [18].

This analysis revealed a significant change in genera populations at T2 samples in AD mice (compared to T0), with an increase in Saccharibacteria, Anaerotruncus, Vampirovibrio, and Alistipes while a reduction in Dorea, Anaerostipes, Hallella, and Ruminococcus 2 was observed (DESeq2 |log2FC|>1 & FDR<0.1).

Importantly, data reported in Fig 2C (right panel) identify a sort of ‘genera signature’ in AD samples (evaluated at 6 months—T2), genera significantly different in AD vs WT mice and consisting in a prevalence of Roseburia, Saccharibacteria, Clostridium, Odoribacter, Bacteroides, and Vampirovibrio, while a consistent decrease in Anaerostipes, Catonella, Ruminococcus, and Shuttleworthia is also evident (DESeq2 |log2FC|>1 & FDR<0.1). The beta diversity of groups compared in Fig 2 was also evaluated and reported in (S1 Fig).

### Gut metabolome profile is altered in 3xTgAD mice

**Fig 3:**
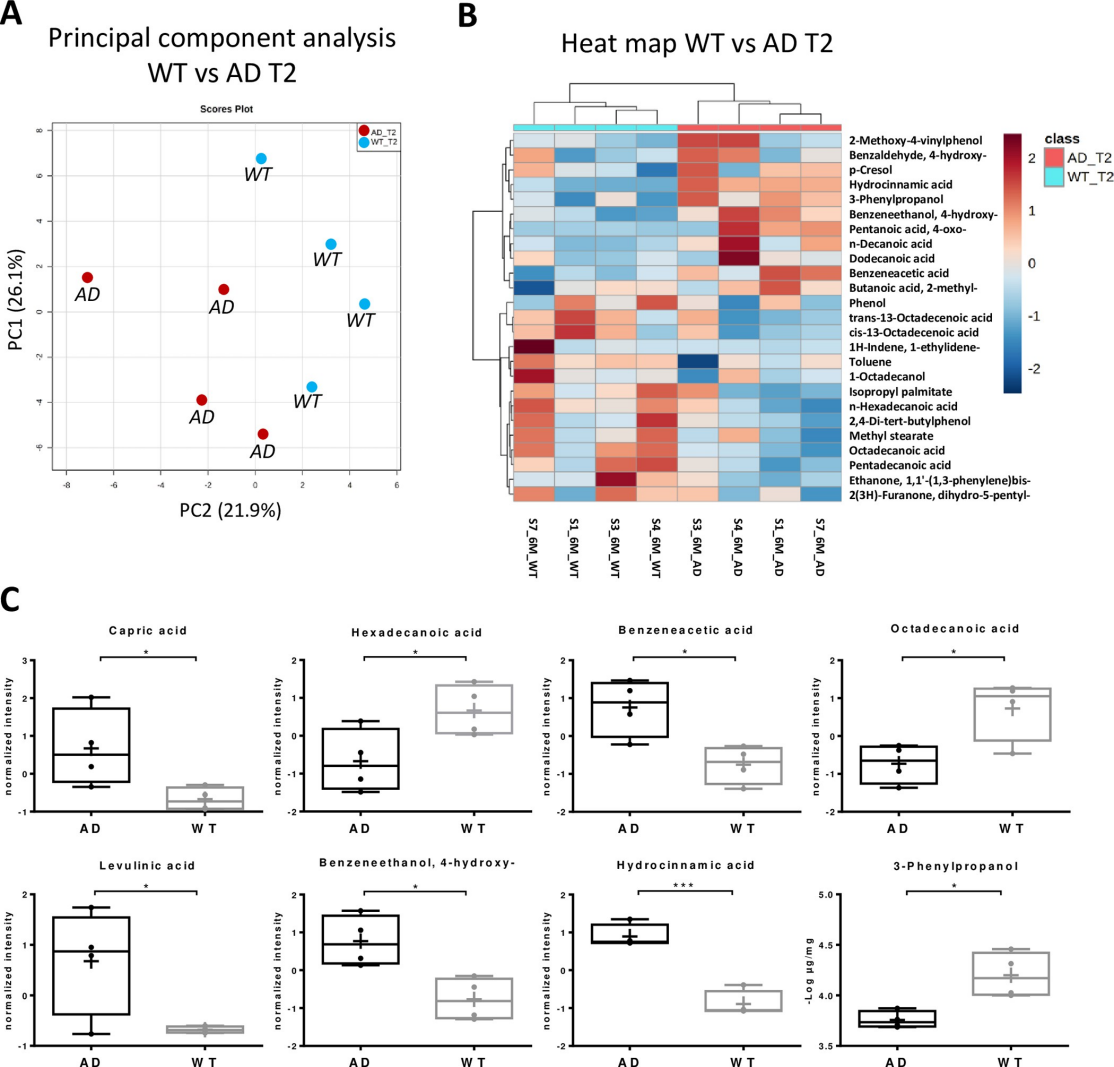
Metabolic profile at six months in AD mouse. A) Score plot of principal component analysis (PCA) showing two distinct groups, the WT (light blue) and the AD mouse model (red). B) Hierarchical clustering heat-map of the most modulated metabolites, C) box plot of modulated molecules at six months (T2), expressed as ‘normalized intensity’, corresponding to normalized abundance of the molecules reported in the heatmap (panel B). * p<0.05; ** p<0.01; *** p<0.001.

Metabolomic analysis of fecal samples showed a strong alteration of intestinal metabolome profile in AD mice, compared to the WT group. A total of 17, 14 and 20 molecules were modulated between the two groups of samples at weaning (T0), two (T1) and six (T2) months, respectively. The hierarchical clustering heatmap and the principal component analysis (PCA) reported in Fig 3A and 3B indicate the presence of a ‘microbiota metabolic signature’ associated to the Alzheimer’s Disease at six months (T2). A detailed list of molecules differently modulated at six months between AD and WT groups is reported in S1 Table. This analysis revealed that Capric, benzeneacetic acid, levulinic, 4 hydroxy benzeneethanol and hydrocinnamic acid were up-regulated in AD mice, while hexadecenoic acid, octadecanoic acid, and 3-phenylpropanol were under-represented (Fig 3C).

**Fig 4:**
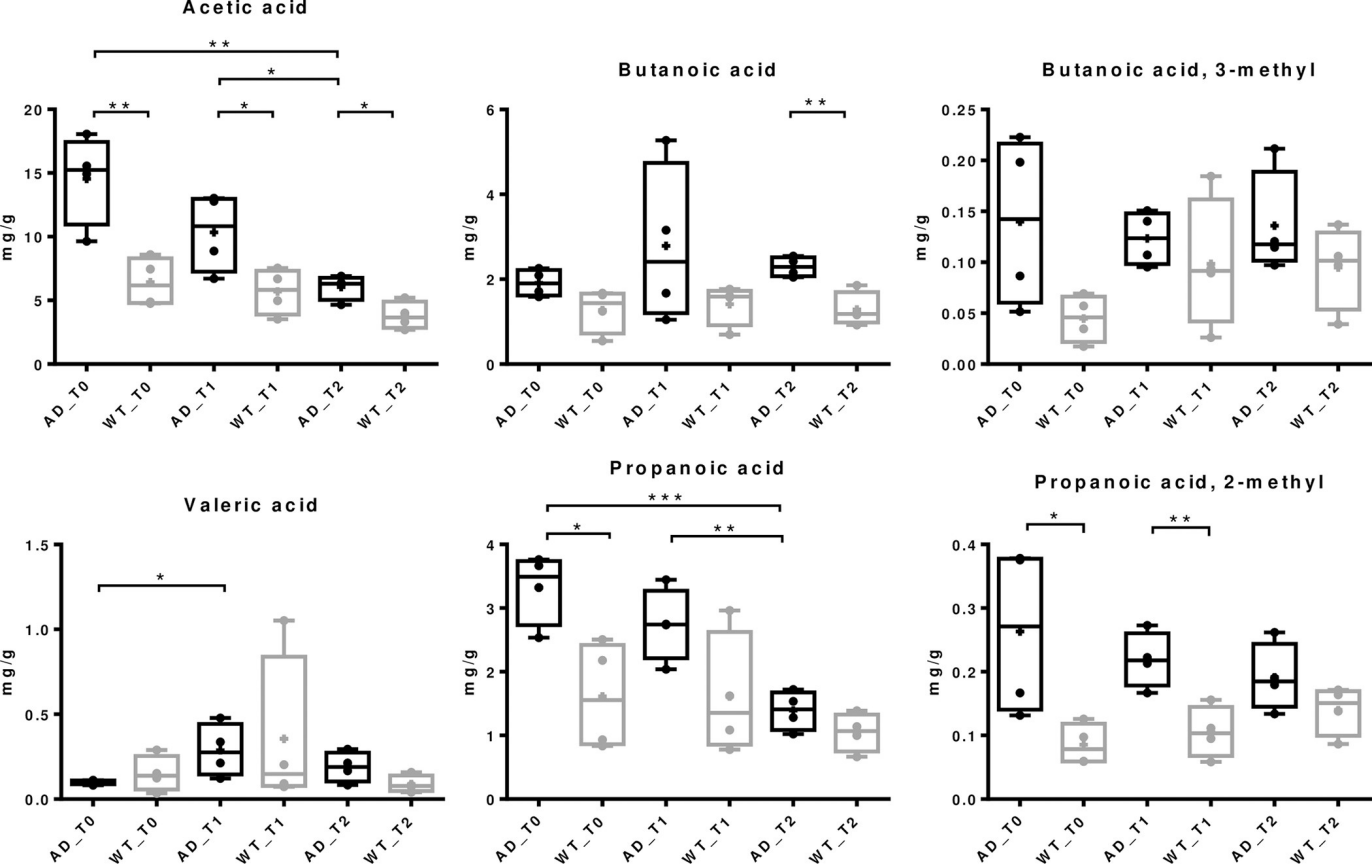
Short-chain fatty acids modulations over time. Box plots with p-values of acetic acid, butanoic acid, 3-methyl butanoic acid, valeric acid, propanoic acid, and 2-methyl propanoic acid at weaning (T0), at two months (T1), and at six months (T2). * p<0.05; ** p<0.01; *** p<0.0005.

Short chain fatty acids (SCFAs) analysis revealed a longitudinal modulation in both AD and WT groups. Indeed, although some significant differences were already present at weaning, such as acetic, propanoic and 2-methyl propanoic acid, and levels of both acetic and butanoic acid are still higher in AD mice at six months, compared to WT, a dramatic and significant decrease of both acetic and propionic acid is however evident in the AD group, in a time-dependent manner (Fig 4). These results suggest that SCFAs abundance changes in the gut microbiota composition may occur in the early stages of the disease, while becoming more evident with the disease progression.

**Fig 5:**
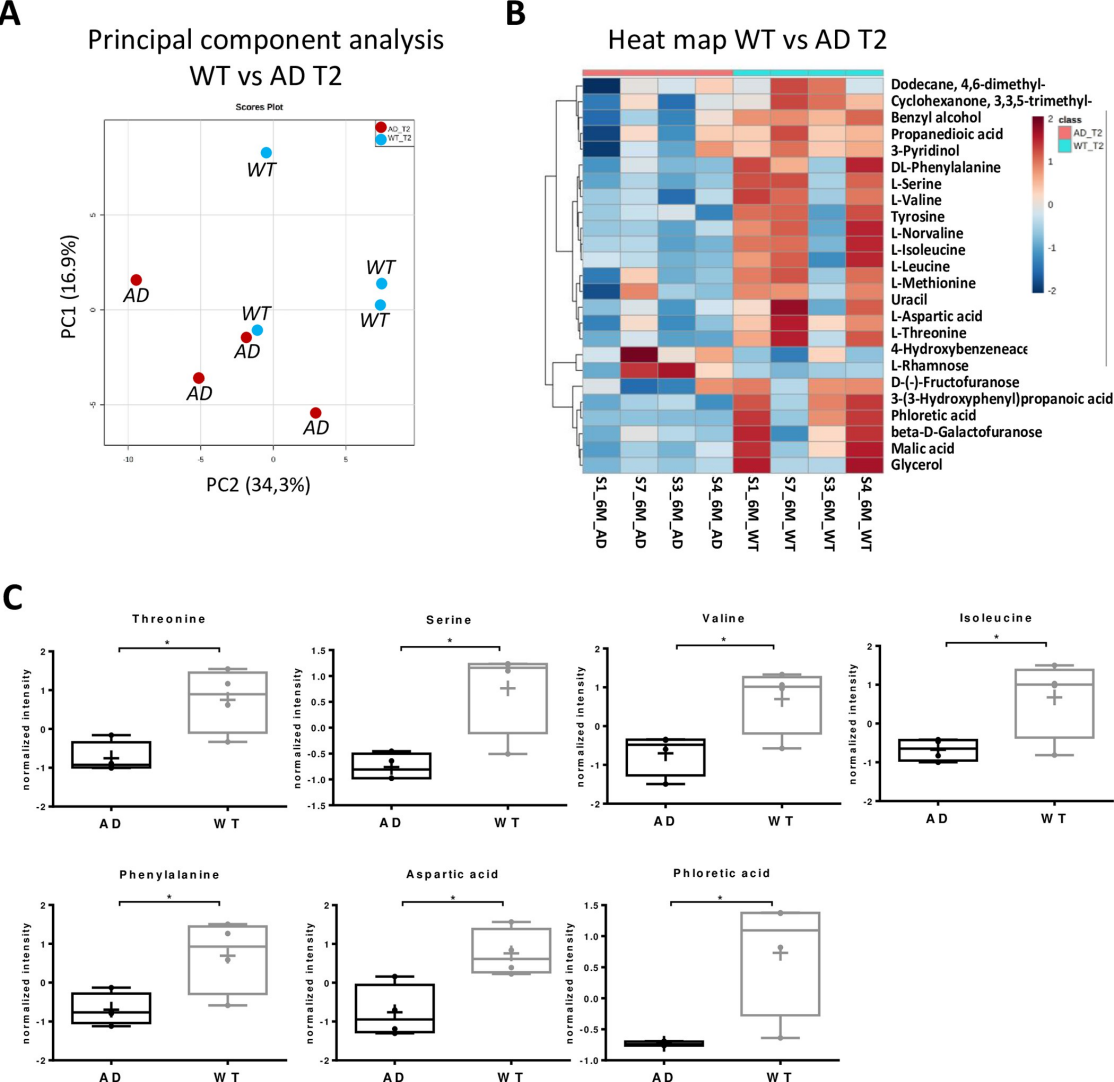
Metabolic profile of small molecules from aqueous phase extraction of AD mouse fecal samples, at six months. A) Score plot of principal component analysis (PCA) showing two distinct groups, the WT (light blue) and the AD mouse model (red). B) Hierarchical clustering heat-map of the most modulated metabolites. C) Box plot of modulated amino acids and phloretic acid at six months (T2), expressed as ‘normalized intensity’, corresponding to normalized abundance of the molecules reported in the heatmap (panel B). * p<0.05; ** p<0.01; *** p<0.0005.

Interestingly, the analysis of polar metabolites performed on the aqueous phase extraction showed an alteration of amino acids levels in the AD group at 6 months, compared to the WT group (Fig 5A and 5B). Indeed, the abundance of threonine, serine, valine, isoleucine, phenylalanine and aspartic acid were lower in stools from AD mice compared to WT matched controls (Fig 5C).

Although to date there are no evidences associated to a lowering of these amino acids level in stools of mouse or human subjects with AD, an alteration of amino acids distribution during the worsening of AD has already been reported in the brain from AD patients [19]. Our findings suggest that a dysregulated ‘gut-brain axis’ might be involved in AD progression [20].

### Intestinal microbiota and metabolome correlations in Alzheimer’s Disease–a Metabologenomic analysis

**Fig 6:**
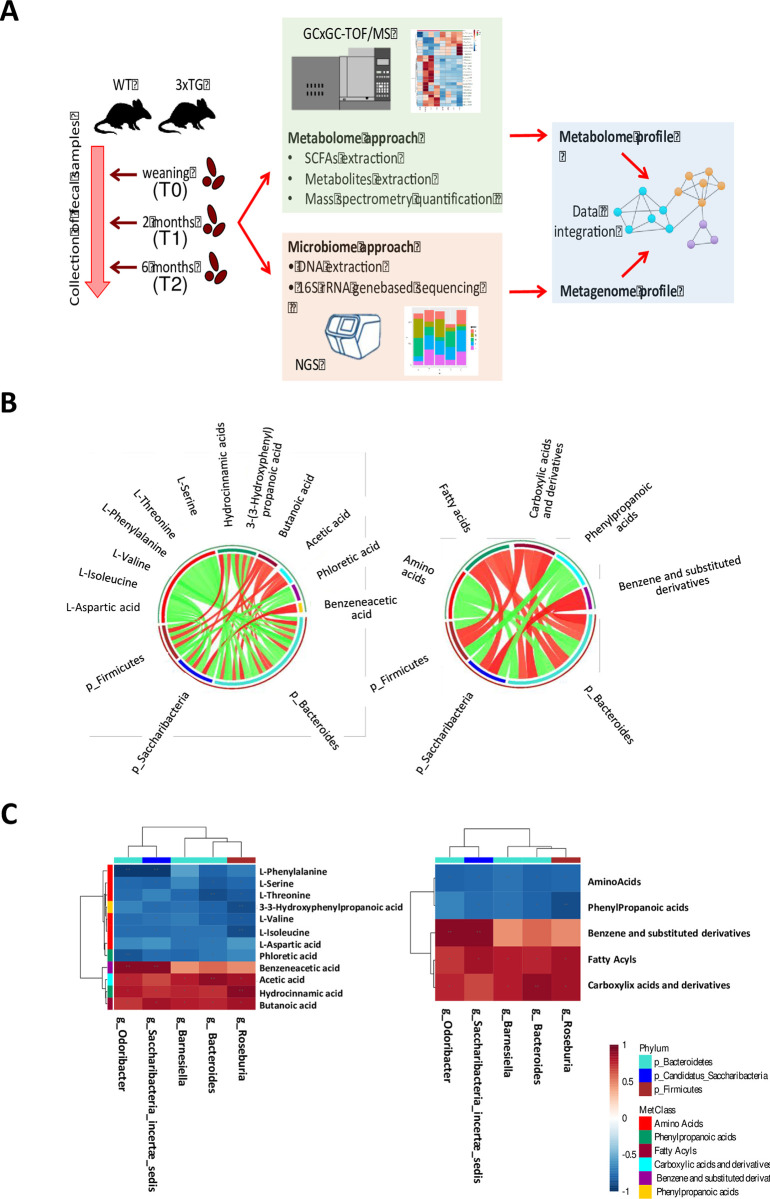
Metabologenomics. A) Workflow of the study. Fecal samples from WT and 3xTG mice at weaning, two and six months were collected and analyzed using metabolomic and metagenomic approaches. Metabolome and metagenome profiles were then analyzed and integrated to explore microbiome variations potentially associated with the progression of the disease. B) Circos plot of Spearman correlation between metabolite-phyla (left panel) and class-metabolites-phyla (right panel). A positive correlation is distinguished by red lines, while a negative correlation by green lines. C) Hierarchical heat maps with Spearman correlation between bacterial genera and metabolites (left panel), and with bacterial genera and metabolites-classes (right panel). The red color indicates a positive correlation, while the blue color indicates a negative correlation. The darker color indicates more statistical significance. Symbol * and ** indicates correlation coefficients smaller than 0.05 or 0.01, respectively.

In order to investigate which bacterial taxa and metabolite-classes or individual metabolite were responsible for the overall associations between gut microbiota and metabolome, the individual correlations between genus-level bacterial abundance profiles, class-level and individual metabolite-level intensity profiles, using the M2IA opensource web server, were performed (Fig 6A). Firstly, the correlations between modulated genera distribution, significant metabolite-classes abundances and their relative individual metabolites (FC>1.3, p-value<0.05) were performed using metagenomic and metabolomic data obtained from the gut analysis at six months. All the data were used for the analysis and no statistical restrictions were used.

Three genera from the Bacteroidetes phylum (Odoribacter, Barnesiella and Bacteroides), a genus from the Candidatus Saccharibacteria phylum (Saccharibacteria genera incertae sedis) and a genus from Firmicutes phylum (Roseburia) showed significant negative correlations with the classes of amino acids and phenylpropanoic acid under-represented in AD at T2, while significant positive correlations were reported with benzene and substituted derivatives, fatty acyls and carboxylic acids (Fig 6B). In the individual metabolite correlation heat map (Fig 6C) the above-mentioned genera showed positive correlations with four metabolites (red) and negative correlations with eight molecules (blue).

**Fig 7:**
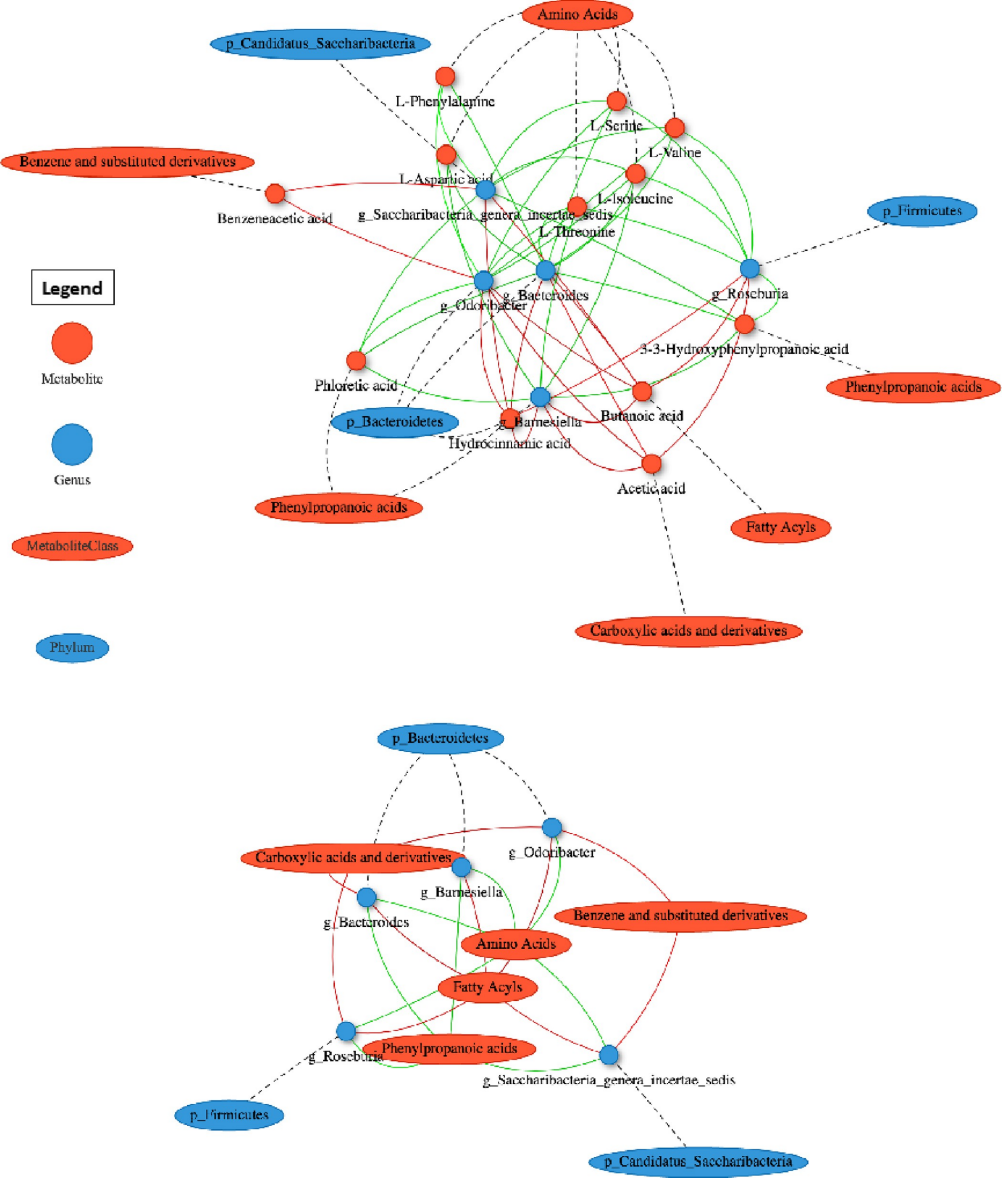
Functional network analysis of microbiota-metabolome correlations. Metabolites and microbes are represented as red circles and blue ovals. Their positive and negative correlations are indicated using red and green lines, respectively. In the upper panel is reported the network correlation between metabolites and genera, while in the bottom panel is reported the functional network between metabolite-classes and phyla.

The complex relationship between microbes and metabolites was then explored by performing a correlation functional network analysis. Five microbes at the genus level belonging to the Firmicutes, Bacteroidetes and Candidatus Saccharibacteria phyla had the most significant correlations with metabolites, as reported in Fig 7. Interestingly, these five genera (Roseburia, Odoribacter, Barnesiella, Bacteroides, and Saccharibacteria incertae sedis) are negatively correlated with amino acids abundances, as well as phloretic and 3-3-hydroxyphenylpropanoic acids, which are characterized by a strong negative correlation with Odoribacter and Roseburia, respectively.

## Discussion

The study of microbiota and, particularly, dysbiosis associated with human diseases is revolutionizing our ideas and modifying the clinical management of many disorders, offering new opportunities to treat patients. Although full development of the individual gut microbiota is achieved within the first 2–3 years of life, with limited fluctuations in its composition during life in healthy individuals, dysbiosis has been documented in several pathological conditions. In particular, gut dysbiosis has been observed in human diseases characterized by gut inflammation such as Celiac Disease, Cystic Fibrosis, Obesity, IBD, diabetes, colon cancer, and more [21–23]. Although whether cause or consequence of disease onset is still under deep investigation, rebalancing gut microbiota by using prebiotics, probiotics, nutraceuticals or fecal transplants seems to have a positive impact on some disease onset/progression, at least in animal models [12,24,25]. Thus, a deep knowledge of gut microbiota modifications associated with specific disorders will help us to better understand the disease and to increase our opportunities to treat patients.

A longitudinal metagenomic and metabolomic analysis of stools samples from a mouse model of AD (3xTgAD), compared to matched WT controls, revealed a dysregulated microbiota and metabolites composition in 6 months-old mice. Our analysis revealed a slight and similar fluctuation of the F/B ratio in both AD and WT groups of mice at weaning and 2 months, while a significant divergence was observed between the groups at 6 months, a time coincident with the progression of the pathology, in this mouse model (S2 Fig) [14]. In parallel, an increase in the Candidatus Saccharibacteria phylum was also found in the microbiota of AD animals at 6 months. Both these data indicate a potential increase in the gut inflammation, previously documented in AD patients [26]. A link between Candidatus Saccharibacteria and patients affected by IBD, potentially correlating with increased gut inflammation, have also been reported [27]. Moreover, in patients affected by AD, an increase in the presence of Proteobacteria was described in the literature, with a similar trend that was also observed in our AD mouse model at 6 months [11].

In line with these results, an increase in the presence of Erysipelotrichaceae, which belongs to the Firmicutes phylum, was also observed in AD mice at 6 months. A similar trend was described in patients affected by IBD [28], which further suggests a potential inflammatory status of the gastrointestinal tract of AD mice, compared to WT, associated with disease progression.

Alpha and beta diversity are useful methods to evaluate the microbiota biodiversity. This analysis revealed a significant difference in biodiversity of WT and AD samples at weaning, while becoming similar at two months, and diverging again at 6 months. The initial divergent biodiversity might be explained since AD and WT mice derived by different mothers housed in different cages. To reduce this potential confounding factor, AD and WT mice were co-housed at weaning. Indeed, alpha and beta diversity were similar in the two groups at 2 months. Therefore, the further divergence in biodiversity observed at 6 months comparing AD and WT animals correlates with disease progression, although whether causative or consequence requires further analysis. Overall, data support the paradigm of a microbiota reshaping in AD mice, upon AD onset. Indeed, synaptic transmission and long-term potentiation (LTP) are already impaired in 3xTgAD mice at 6 months, while intracellular Aβ accumulation is detected in brain regions as early as 3–4 months.

In parallel, studying the microbiota-associated metabolome increases our knowledge of the complex and mutual crosstalk between the intestinal flora and host. Indeed, the activity of gut microbiota components is fundamental for the digestion of food, production of critical essential elements such as some amino acids but also, importantly, producing compounds able to contribute to the control of gut epithelia growth, cell differentiation/proliferation, tissue permeability, inhibition of gut inflammation through direct/indirect (by means of immune cells) activity [29]. Although the precise molecular events and mechanisms at the base of these plethora of gut microbiota activities are still elusive and under deep investigation, the role of few molecules such as short-chain fatty acids (SCFA), long-chain fatty acids (LCFA) and amino acids is emerging [30]. Therefore, the characterization and evaluation of the activity of these molecules on host tissues is very important. Particularly, SCFA have been observed to exert a positive impact on gut inflammation, through their ability in modulating the Treg component of the immune system [31]. In line with this, gut proinflammatory diseases are frequently paralleled by a progressive decrease in gut SCFA abundance [32]. Although a higher level of few SCFA in AD mice at six months, such as acetic and butanoic acid, are apparently discording with the latter concept and with our speculations reported above, it is important to note the drastic longitudinal decrease of both acetic and propanoic acid in AD mice, while no major differences were observed in the WT group within 6 months.

Our analysis also evidenced a significantly decreased concentration of an amino acid group in samples from AD mice at 6 months compared to WT matched controls, some of which have been reported to be potentially linked to gut inflammation, such as phenylalanine, isoleucine and valine [33]. Although the meaning in these changes is still not completely clear, it might be linked to an altered gut-brain axis circuit.

Finally, an integrated analysis combining both metagenomic (16S) and metabolomic data was recently introduced (metabologenomics), with the aim of correlating the modulation of some metabolites to a corresponding modulated component of the intestinal ecosystem [13]. Studying AD from a metabologenomics perspective can provide new insights into AD pathogenesis and may lead to the discovery of dietary probiotics and metabolite supplements that could delay AD progression. Our results not only identified perturbations of the gut microbiome in preclinical stages of the disease, which suggested the potential role of microbiota in modulating the pathogenesis of AD, but it also allowed the discovery of new relationships between bacteria and metabolites. Importantly, the integration of these data revealed the potential involvement of phloretic and 3-3-hydroxyphenylpropanoic acids, also called desaminotyrosine (DAT) and dihydro-3-coumaric acid, which are well-known metabolites produced by microbiota enteric bacteria [34], and that were down-regulated at 6 months. High gut levels of DAT have been already associated to immune-regulatory functions [35], while Wang and colleagues demonstrated that 3-3-hydroxyphenylpropanoic acid might interfere with the assembly of β-amyloid (Aβ) peptides into neurotoxic Aβ aggregates and could play key roles in AD pathogenesis [36].

## Conclusions

In conclusion, our observations suggest that the intestinal microbiota could influence the onset and/or progression of Alzheimer’s Disease neurodegeneration.

In addition, although the most relevant differences in the metabolic profile become evident at six months of age in 3xTgAD mice, SCFA and biodiversity alterations appeared earlier, suggesting that a perturbation of microbiome can occur before the appearance of the main clinical signs of the disease.

## Supporting information

S1 Fig AD progression drives global perturbations of gut microbiota. Differences at the genus taxonomic rank in mice stool microbiota between T 2 and T 0 time points (A) for AD samples between T 1 and T 2 time points (B) for AD samples and between WT samples and AD samples at T 2 time point (C). Plots show beta diversity values in a PCA diagram.
(PDF)

S2 Fig Aβ immunoreactivity in 3 xTg AD. Aβ immunoreactivity in 3 xTg AD (left figure) mice compared to wild type mice (right figure) Low magnification view of prefrontal cortex (A) hippocampus (B) basolateral amygdala (C) from 8 months old mice following staining with 6 E 10 specific antibody. Higher magnification views of section (D-E) showing intraneuronal Aβ immunoreactivity. Original magnifications 5x (A-C), 20x (D-F). Images were acquired using a Neurolucida microscope.
(PDF)

S1 Table Main molecules modulated at six months. (PDF)
