# Supplementary material for: A Metabologenomic approach reveals alterations in the gut microbiota of a mouse model of Alzheimer’s disease
Source: PLoS One. 2022 Aug 24;17(8):e0273036. doi: 10.1371/journal.pone.0273036 (PMC9401139; doi:10.1371/journal.pone.0273036)
Supplement: S1 Fig — Differences at the genus taxonomic rank in mice stool microbiota between T 2 and T 0 time points (A) for AD samples between T 1 and T 2 time points (B) for AD samples and between WT samples and AD samples at T 2 time point (C). Plots show beta diversity values in a PCA diagram. (PDF) [file pone.0273036.s001.pdf]

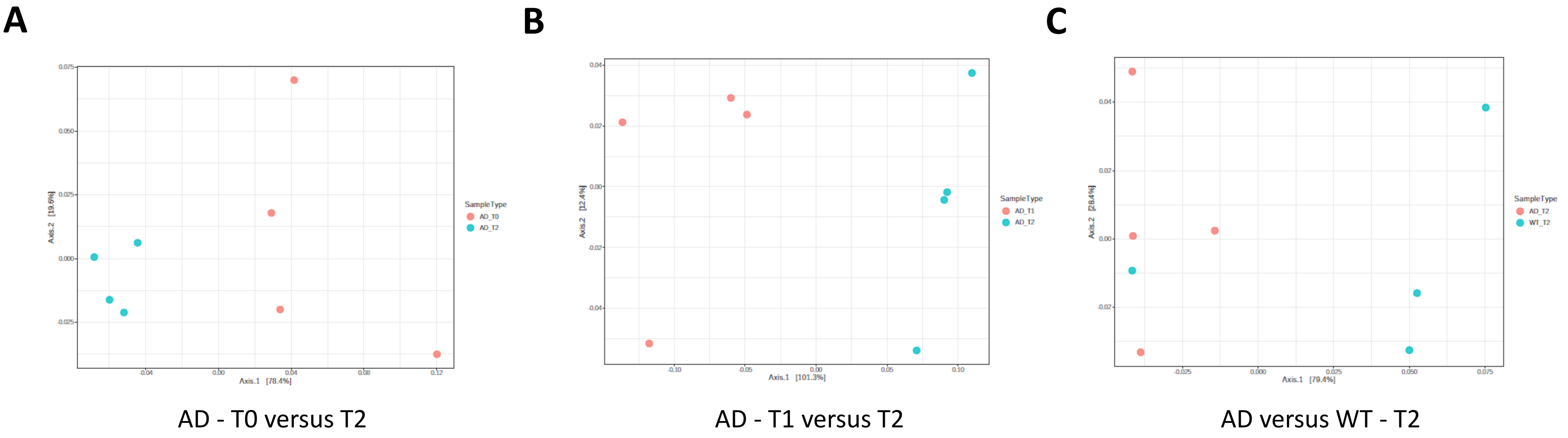

SUPPLEMENTARY FIGURE 1: AD progression drives global perturbations of gut microbiota. Differences at the genus taxonomic rank in mice stool microbiota between T2 and T0 time points (A), for AD samples; between T1 and T2 time points (B), for AD samples and between WT samples and AD samples at T2 time point (C). Plots show beta diversity values in a PCA diagram.
