# Supplementary material for: A Metabologenomic approach reveals alterations in the gut microbiota of a mouse model of Alzheimer’s disease
Source: PLoS One. 2022 Aug 24;17(8):e0273036. doi: 10.1371/journal.pone.0273036 (PMC9401139; doi:10.1371/journal.pone.0273036)
Supplement: S2 Fig — Aβ immunoreactivity in 3 xTg AD (left figure) mice compared to wild type mice (right figure) Low magnification view of prefrontal cortex (A) hippocampus (B) basolateral amygdala (C) from 8 months old mice following staining with 6 E 10 specific antibody. Higher magnification views of section (D-E) showing intraneuronal Aβ immunoreactivity. Original magnifications 5x (A-C), 20x (D-F). Images were acquired using a Neurolucida microscope. (PDF) [file pone.0273036.s002.pdf]

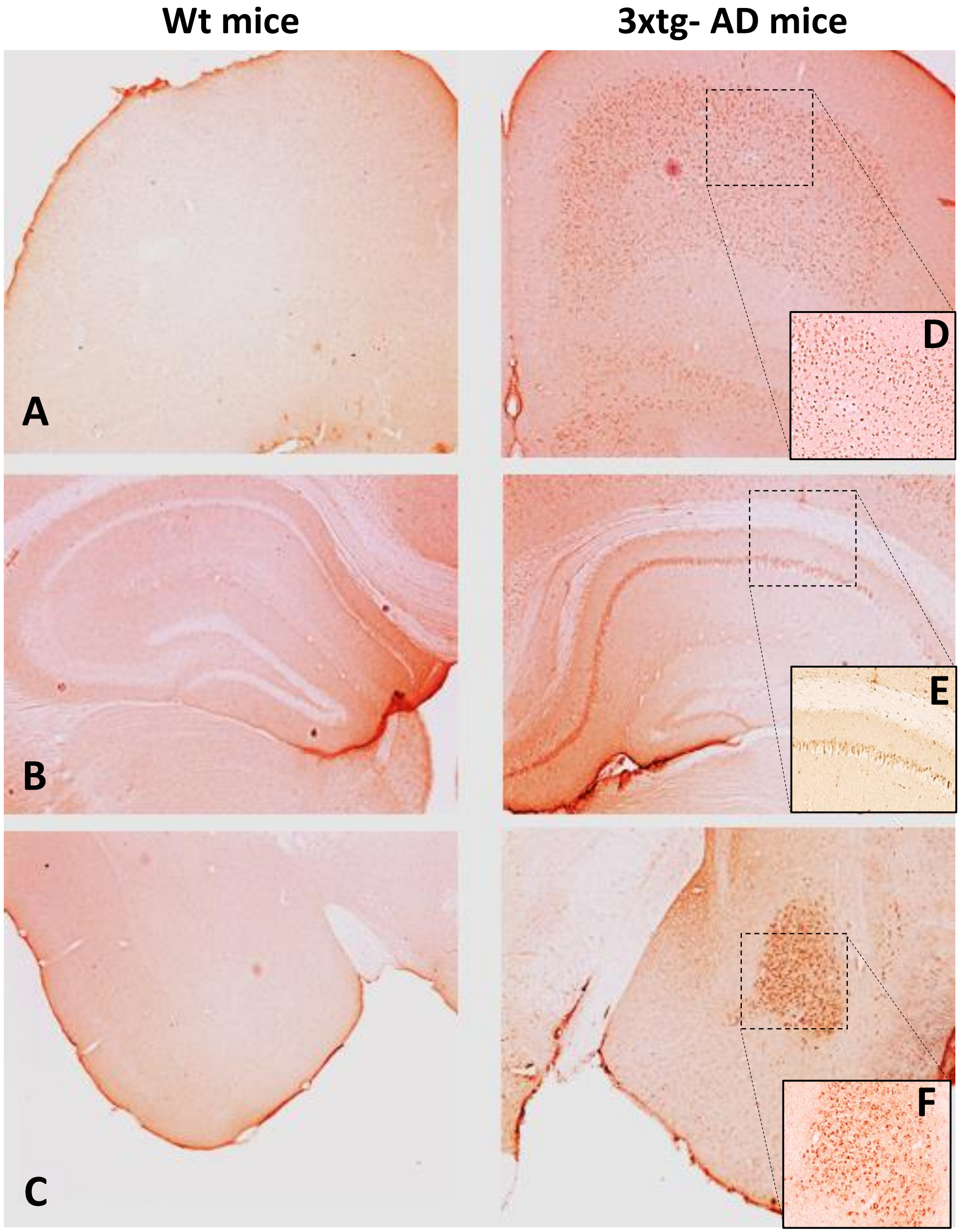

SUPPLEMENTARY FIGURE 2. Aβ immunoreactivity in 3xTg-AD (left figure) mice compared to wild type mice (right figure).
Low-magnification view of prefrontal cortex (A), hippocampus (B) , basolateral amygdala (C) from 8 months old mice following staining with 6E10 -specific antibody.
Higher-magnification views of section (D-E) showing intraneuronal Aβ immunoreactivity.
Original magnifications: 5 (A,B,C), 20 (D, E, F).
Images were acquired using a Neurolucida microscope**.**

Method.

For immunohistochemical studies, mice were anesthetized with isoflurane and transcardially perfused with paraformaldehyde (4% in 0.1 M phosphate buffer, pH 7.4). Sections from the Prefrontal cortex, hippocampus and basolateral amygdala (35 µm thick) were coronally cut on a microtome. Free-floating sections were incubated overnight with Aβ primary antibodies (anti-β-Amyloid, 6E10 **,** 1:1000, Biolegend).

After washing, sections were incubated in proper biotinylated secondary antibodies. For visualization, the avidin–peroxidase protocol (ABC, Vector Laboratories, UK) was applied, using DAB (Sigma-Aldrich, Milan, Italy) as chromogen. After washing, the sections were mounted on slides, air-dried, dehydrated in ascending concentrations of ethanol, and cleared with xylene.
