# Supplementary material for: A Metabologenomic approach reveals alterations in the gut microbiota of a mouse model of Alzheimer’s disease
Source: PLoS One. 2022 Aug 24;17(8):e0273036. doi: 10.1371/journal.pone.0273036 (PMC9401139; doi:10.1371/journal.pone.0273036)
Supplement: S1 Table — (PDF) [file pone.0273036.s003.pdf]

Table 1. Main molecules modulated at six months. Name, HMDB id, fold change, p-value and relative classes are listed.

	Name	*HMDB ID*	*FC*	*p value*	*Class*
UNTARGETED AQUEOUS PHASE	Benzyl alcohol	HMDB0003119	0.814	0.0054	Benzene and substituted derivatives
	L-Serine	HMDB00187	0.400	0.0138	Amino Acids
	L-Aspartic acid	HMDB00191	0.613	0.0148	Amino Acids
	L-Threonine	HMDB00167	0.639	0.0162	Benzene and substituted derivatives
	3-3-Hydroxyphenylpropanoic acid	HMDB0124925	0.260	0.0171	Phenylpropanoic acids
	Phloretic acid	HMDB02199	0.053	0.0216	Benzene and substituted derivatives
	L-Valine	HMDB00883	0.411	0.0335	Amino Acids
	DL-Phenylalanine	HMDB00159	0.502	0.0347	Amino Acids
	Cyclohexanone, 3,3,5-trimethyl-	HMDB0031195	0.862	0.0353	Organooxygen compounds
	L-Isoleucine	HMDB00172	0.638	0.0427	Amino Acids
UNTARGETED ORGANIC PHASE	Hydrocinnamic acid	HMDB0000764	8.114	0.0002	Phenylpropanoic acids
	Benzeneethanol, 4-hydroxy-	HMDB0004284	2.921	0.0122	Phenols
	Benzeneacetic acid	HMDB0000209	1.869	0.0151	Benzene and substituted derivatives
	Octadecanoic acid	HMDB00827	0.769	0.0228	Fatty Acyls
	3-Phenylpropanol	HMDB0033962	1.993	0.0232	Benzene and substituted derivatives
	Levulinic acid	HMDB0000720	3.607	0.0413	Keto acids and derivatives
	Decanoic acid	HMDB00511	1.583	0.0447	Fatty Acyls
	Hexadecanoic acid	HMDB00220	0.839	0.0461	Fatty Acyls
SCFAs	Butanoic acid	HMDB00039	1.782	0.0048	Fatty Acyls
	Acetic acid	HMDB00042	1.591	0.0213	Carboxylic acids and derivatives
